# Convolutional Neural Network Approaches in Median Nerve Morphological Assessment from Ultrasound Images

**DOI:** 10.3390/jimaging10010013

**Published:** 2024-01-05

**Authors:** Shion Ando, Ping Yeap Loh

**Affiliations:** 1 Department of Mechanical Engineering, Faculty of Engineering, Kyushu University, Fukuoka 819-0395, Japan; ando-shion@mech.kyushu-u.ac.jp; 2 Department of Human Life Design and Science, Faculty of Design, Kyushu University, Fukuoka 819-0395, Japan

**Keywords:** carpal tunnel syndrome, U-Net, SegNet, semantic segmentation

## Abstract

Ultrasound imaging has been used to investigate compression of the median nerve in carpal tunnel syndrome patients. Ultrasound imaging and the extraction of median nerve parameters from ultrasound images are crucial and are usually performed manually by experts. The manual annotation of ultrasound images relies on experience, and intra- and interrater reliability may vary among studies. In this study, two types of convolutional neural networks (CNNs), U-Net and SegNet, were used to extract the median nerve morphology. To the best of our knowledge, the application of these methods to ultrasound imaging of the median nerve has not yet been investigated. Spearman’s correlation and Bland–Altman analyses were performed to investigate the correlation and agreement between manual annotation and CNN estimation, namely, the cross-sectional area, circumference, and diameter of the median nerve. The results showed that the intersection over union (IoU) of U-Net (0.717) was greater than that of SegNet (0.625). A few images in SegNet had an IoU below 0.6, decreasing the average IoU. In both models, the IoU decreased when the median nerve was elongated longitudinally with a blurred outline. The Bland–Altman analysis revealed that, in general, both the U-Net- and SegNet-estimated measurements showed 95% limits of agreement with manual annotation. These results show that these CNN models are promising tools for median nerve ultrasound imaging analysis.

## 1. Introduction

**Figure 1:**
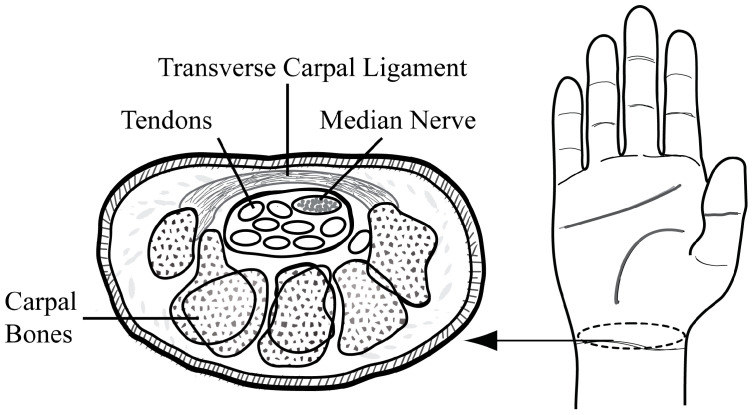
Cross-sectional view of the carpal tunnel.

Carpal tunnel syndrome (CTS) is one of the most commonly reported peripheral nerve entrapment syndromes of the upper limb and is characterized by symptomatic compression neuropathy of the median nerve at the level of the wrist, accompanied by increased pressure within the carpal tunnel and decreased function of the nerve at that level [1]. As illustrated in Figure 1, the median nerve passes through the carpal tunnel, a confined space, and is vulnerable to compression stress from intra-tunnel pressure and the surrounding structures such as the flexor tendons and transverse carpal ligament. Previous studies have shown that individual or combined dynamic finger and thumb movements impose compressive stress on the median nerve owing to the gliding motion of the tendons, which causes them to deform [2,3,4,5].

Various imaging techniques, such as ultrasound (US), magnetic resonance imaging (MRI), and computed tomography (CT), have been used to understand carpal tunnel anatomy and the characteristics of the median nerve in healthy individuals and patients with CTS [6,7,8]. MRI has demonstrated the ability to detect pathological changes in the median nerve and has revealed bowing of the transverse carpal ligament (TCL) in patients with CTS [9,10,11]. However, MRI and CT have several limitations, such as difficult-to-meet requirements for the rooms that house the machines, high costs, slow complete scan speeds, and contraindications in patients that can prevent imaging. Nonetheless, recent studies have successfully incorporated robot-assisted US techniques to acquire three-dimensional representations of carpal tunnel morphology and have demonstrated their validity and reliability for reconstructing the internal surface of the carpal tunnel, thereby enabling a detailed investigation of the spatial relationships between the median nerve and its surrounding structures [12,13,14].

By contrast, to understand the morphological and biomechanical characteristics of the median nerve and surrounding anatomical structures, such as finger flexor tendons, subsynovial connective tissues, and blood circulation in and around the median nerve, high-resolution US imaging is a more convenient method for carpal tunnel examination [15,16,17,18,19,20]. The advantages of high-resolution US imaging include noninvasive, dynamic, and real-time imaging; portable machine size; and inexpensive and easy methods that can be used to investigate the behavior of the median nerve during dynamic changes of the wrist and finger joints [21,22]. Moreover, it is possible to monitor acute changes in the morphological characteristics of the median nerve [23,24,25].

In recent years, deep learning (DL) techniques, such as convolutional neural networks (CNNs), have been applied in US image analysis [26,27,28]. CNNs are particularly useful in semantic segmentation for extracting labeled images at the pixel level [29]. Given their capacity to learn intricate patterns and structures from large volumes of data, deep learning algorithms can be trained to recognize subtle changes in median nerve morphology that may be overlooked by the human eye. Additionally, the automated nature of deep learning models can significantly expedite the process of image analysis, potentially making US a more feasible and efficient tool for understanding the dynamic movement within the carpal tunnel.

U-Net is commonly used as a segmentation method in medical imaging. U-Net is a CNN with an encoder–decoder structure (see Appendix A). The encoder extracts a feature map from the image, and the decoder creates a segmented high-resolution image. Since it was proposed by Ronneberger et al. [30], U-Net has been widely used for the semantic segmentation of medical images [31,32]. SegNet is also a CNN with an encoder–decoder architecture. It has been developed mainly for road scene material segmentation [30]. In contrast to U-Net, SegNet uses unpooling instead of deconvolution in the decoder, which reduces the number of parameters required for learning. Consequently, learning time and memory capacity can be reduced [31]. For instance, Xin et al. applied U-Net to flexor digitorum superficial US images [32]. They reported that the average intersection over union (IoU) was 84.5%, which was higher than that of manual annotation by junior physicians (60.7%). A lesion in the breast was segmented using a modified U-Net, which yielded an IoU of 80.6% [33]. In addition, Singh et al. applied various CNNs for breast tumor segmentation. According to these authors, the mean IoUs of U-Net and SegNet were 77.0 and 51.0%, respectively [34]. Recently, Vianna et al. studied the accuracy of U-Net and SegNet for US-based breast lesion segmentation. Although they did not report the IoU, the DICE coefficients of U-Net and SegNet were 86.3 and 81.1%, respectively, indicating the superiority of U-Net [35]. These findings highlight the capabilities of these models in handling intricacies and challenges specific to ultrasound imaging in medical applications.

In this study, we hypothesized that employing U-Net and SegNet deep learning models for the extraction of median nerve morphology would lead to superior accuracy. The veracity of this hypothesis was evaluated by comparing the congruence of the measurements of median nerve parameters. Therefore, the objective of this study was to apply U-Net and SegNet to extract median nerve morphology and assess their accuracy. To achieve this objective, the median nerve cross-sectional area (MNCSA), circumference, and diameter were calculated and compared using manual annotation.

## 2. Materials and Methods

### 2.1. Ultrasound Image Acqusition and Dataset Preparation

Ultrasound images of the median nerve were obtained from twelve healthy right-handed participants in a previous study [25]. US images of the median nerve at the proximal carpal tunnel were acquired using a LOGIQ e ultrasound system (GE Healthcare, Milwaukee, WI, USA) with a 12 L-RS transducer and an imaging frequency bandwidth set at 12 MHz. During the ultrasound exam, the examiner (P.Y.L.) positioned the ultrasound transducer on the sonar pad, ensuring that too much pressure was not exerted on the wrist. The forearm was laid out in the supine position and supported by an arm resting on a table, with the elbow bent at a 30-degree angle. For accurate identification of the median nerve in the transverse plane, the examiner aligned the ultrasound transducer with the distal wrist crease, using the proximal edge of the pisiform bone as a consistent anatomical reference point under all conditions. In the experiment [25], participants participated in three sessions of trials, namely, the control (seven time blocks) and typing tasks I and II (eight time blocks, with each time block lasting for 30 min). Three US images were acquired from each wrist at the end of every 30 min time block in all conditions. In this study, a total of 1080 images were accumulated from three sessions.

From the aforementioned acquired US images, a total of 600 median nerve images were randomly selected to serve as the dataset for this study. The dataset included images of the left and right wrists because the main objective was to evaluate the application of U-Net and SegNet in the extraction of median nerve morphology. The original images were in TIFF format and had a resolution of 532 × 434. In the RAW datasets, the images include the outer frame. If these images were used as inputs, the size of the input would increase, which increases the training parameters and calculation cost. Thus, the outer frame was removed, and the size of the input image was set to 300 × 300. Next, the images were resized to 256 × 256 using the OpenCV library (version 3.1). The images were randomly categorized into 450 images (75.0%) for training, 50 images (8.3%) for validation, and 100 images (16.7%) for testing. Furthermore, the training images were augmented to create 900 images by randomly flipping them in the vertical and horizontal directions. Because semantic segmentation is a supervised learning method, labeled ground-truth images are needed. Therefore, the median nerve of all the images were manually labeled. The accuracy of the CNNs was evaluated by comparing the labeled ground-truth images with the output images of U-Net and SegNet.

### 2.2. Manual Annotation of the Median Nerve

**Figure 2:**
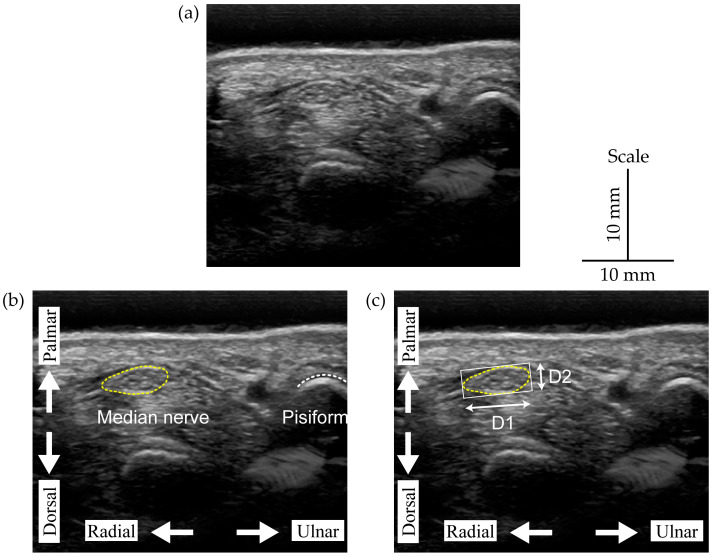
(**a**) Original image, (**b**) manual annotation of the MNCSA, (**c**) median nerve diameter annotation using the minimum bounding rectangle method.

The median nerve was identified at the superficial level by its hypoechogenic rim, which includes the hypoechogenic nerve fascicles, whereas the extraneural boundary was identified by its hyperechogenic appearance. The MNCSA and circumference were measured via a tracing method using ImageJ software (v1.51). This involved outlining the boundary between the hypoechoic interior of the median nerve and the hyperechogenic epineurium [36]. Subsequently, the minimum bounding rectangle method using the OpenCV library was used to quantify the longest diameters in the radial–ulnar (D1) and dorsal–palmar (D2) directions on the traced outline of the median nerve [25], as shown in Figure 2. The diameters (D1 and D2) of the median nerve area masked by the CNN were measured using the same minimum bounding rectangle method.

### 2.3. Deep Learning Estimation of the Median Nerve

**Figure A8:**
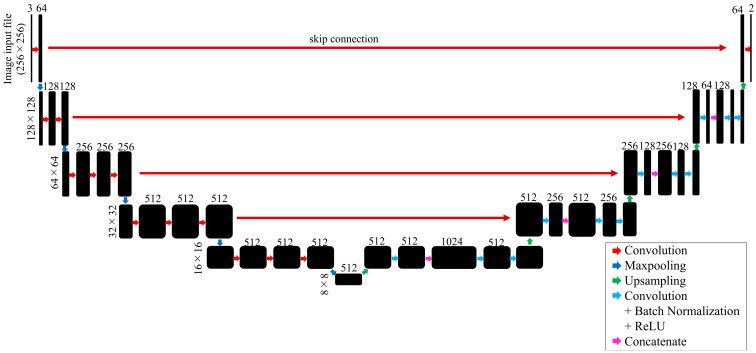
Architecture of U-Net.

**Figure A9:**
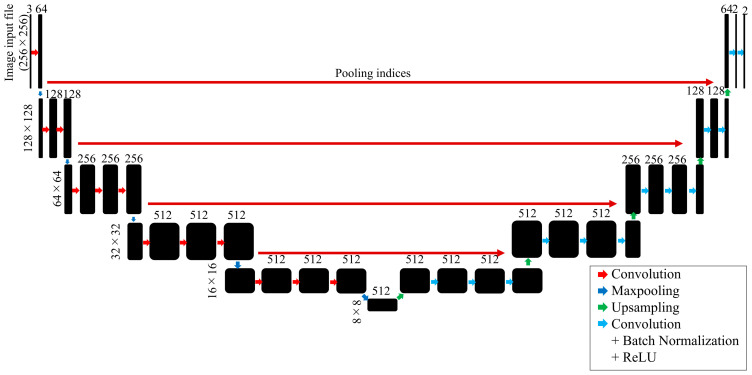
Architecture of SegNet.

In this study, image segmentation was performed using U-Net and SegNet. Because these architectures are described in detail in Appendix A, only the outline is mentioned here. Figure A8 and Figure A9 show the architectures of U-Net and SegNet, respectively. Both constitute an encoder (contracting process) and a decoder (expanding process). The encoder of U-Net has 2 × 2 convolution and 3 × 3 max-pooling layers, which produce an 8 × 8 feature map. The decoder produces a 256 × 256 output image with deconvolution and convolution layers. Like U-Net, SegNet has an encoder that includes convolution and max-pooling layers. On the other hand, its decoder has unpooling layers, which is an architecture different from that of U-Net.

The ReLU activation function was applied for the convolution layer, thereby alleviating the vanishing gradient problem and reducing the calculation cost. Adam was selected as the optimization algorithm. Binary cross-entropy was employed for the loss function. All the source code was written in Python 3.8.12 and executed on a laptop computer with an 11th Gen Intel^®^ Core ^®^ i7-1195G (Intel, Santa Clara, CA, USA) as the CPU, 16 GB of RAM, and Intel^®^ Iris^®^ Xe Graphics (Intel, Santa Clara, CA, USA) as the GP. The keras and TensorFlow versions were 2.4.3 and 2.3.0, respectively.

### 2.4. Statistical Analysis

The relationships between the MNCSA, circumference, and diameter (D1 and D2) measurements obtained through manual annotation and the CNNs were evaluated using Spearman’s correlation coefficient. This analysis aims to identify and measure the level of association between manual annotations and those obtained from a CNN (U-Net or SegNet). The segmentation accuracies of U-Net and SegNet were compared and evaluated using Student’s *t*-test. The agreement of all measurements obtained by manual annotation with those obtained by U-Net and SegNet was then assessed using Bland–Altman analysis. The aim was to assess the agreement and identify any systematic bias between manual annotations and CNNs. All the statistical analyses were performed using SPSS version 26.0 software (IBM Corp., Armonk, NY, USA). All the results are presented as the mean ± standard deviation (S.D.) unless otherwise specified. The significance level was set at *p* < 0.05.

## 3. Results

### 3.1. U-Net and SegNet Analysis

**Figure 3:**
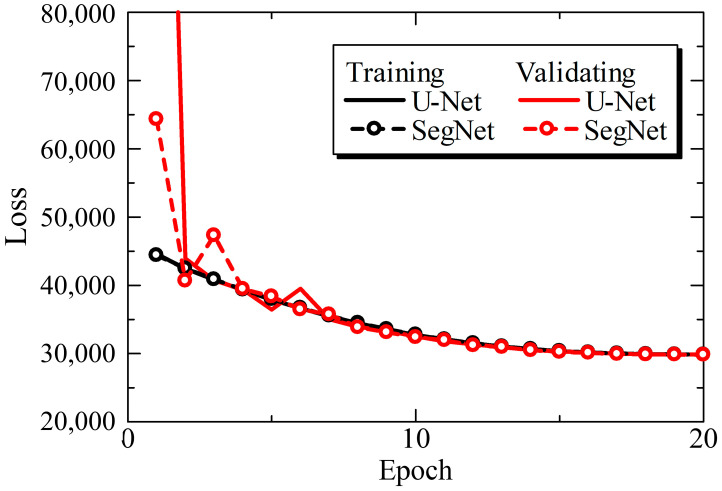
Training and validating losses of U-Net and SegNet.

First, the learning progress was investigated for U-Net and SegNet. Figure 3 shows the learning curves for each model. The training and validation datasets were divided into five batches. In both models, the loss converged when the number of epochs was 20. Therefore, the number of epochs was set to 20. The differences in loss between the models at 20 epochs were 0.05% and 0.15% for the training and validation datasets, respectively, which are sufficiently small. Furthermore, the loss decreased monotonically, indicating that overfitting did not occur in the models.

Subsequently, the model accuracies of U-Net and SegNet were evaluated. For quantitative evaluation, several indicators, i.e., precision, recall, DICE, and IoU, were introduced. These are defined as follows:

$$Precision\equiv \frac{TP}{TP+FP}$$

$$Recall\equiv \frac{TP}{TP+FN}$$

DICE≡21Precision+1Recall

$$IoU\equiv \frac{TP}{TP+FP+FN}$$

where TP is the number of true positives, FP is the number of false positives, TN is the number of true negatives, and FN is the number of false negatives. TP represents the area that should be in the object and is actually in the object. Similarly, TN represents the area classified as background in both the ground truth and CNN images. FP represents the area that should be in the object but is labeled as background. Finally, FN represents the area that should be in the background but is classified as part of the object.

**Figure 4:**
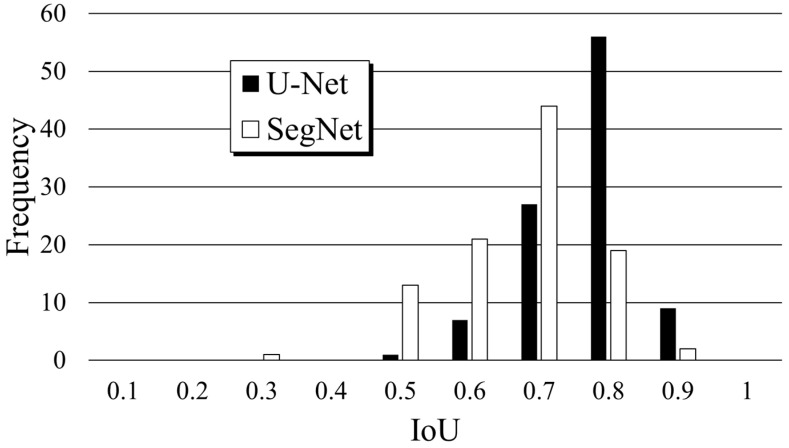
Distributions of the IoU results of U-Net and SegNet.

Table 1 lists the mean and S.D. of the precision, recall, DICE, and IoU of U-Net and SegNet. In addition, a *t*-test was carried out to determine whether the differences in these metrics between U-Net and SegNet were statistically significant. As a result, the *p*-values of these metrics between U-Net and SegNet were almost 1 × 10^−11^, i.e., sufficiently lower than 5%. Thus, the differences were statistically significant. The precision was greater for U-Net than for SegNet, which revealed that FP was lower for U-Net. In contrast, the recall was slightly greater for SegNet, indicating that FN was lower for SegNet. DICE is the harmonic mean of precision and recall. Because the difference between U-Net and SegNet in terms of recall was relatively small, the difference in accuracy (DICE) was greater for U-Net. The IoU is the most important metric because it indicates how accurately the median nerve is predicted. The mean IoU of U-Net was 0.091 larger than that of SegNet. Figure 4 shows the frequency of the IoU results. The mode values were 0.8 and 0.7 for U-Net and SegNet, respectively, and the distribution of the IoU results of U-Net was greater than that of SegNet. The smallest values were 0.498 and 0.231 for U-Net and SegNet, respectively, which increased the mean IoU for U-Net.

**Figure 5:**
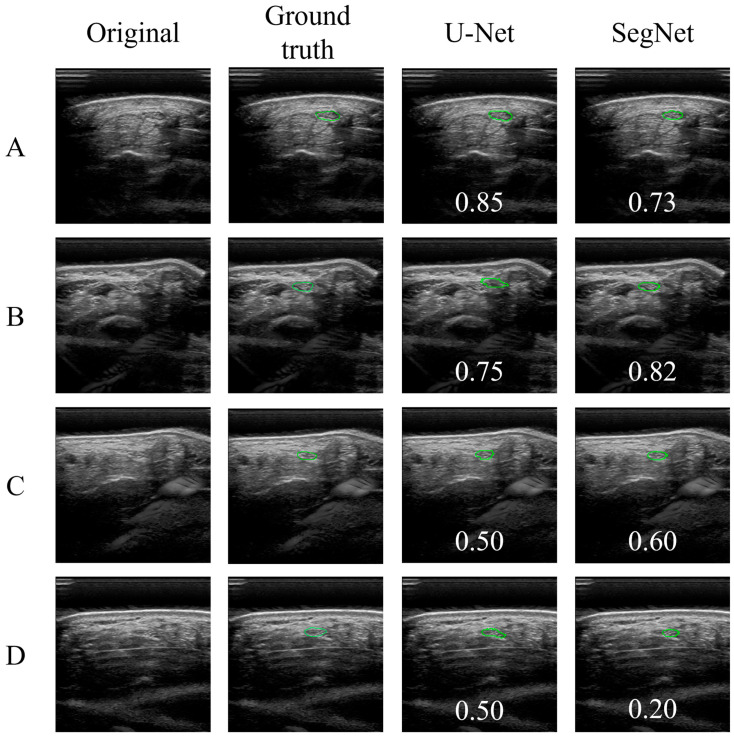
Examples of the original, ground truth, and output images obtained from U-Net and SegNet. Green outline shows median nerve. The numbers indicate the IoU.

Figure 5 shows examples of the ground truth, output data, and IoU. For images A and B, both U-Net and SegNet captured the outline of the median nerve (green outline) well, and the IoU was relatively large. In contrast, images C and D had relatively small IoUs. For image D, the median nerve area of the SegNet output images was significantly smaller than that of the ground truth. In general, compressive stress influences median nerve elongation. When the median nerve was elongated and its outline became blurred, the IoU value was small.

### 3.2. Correlation and Agreement of Manual Annotation and CNN

Next, the accuracy of the CNNs in extracting the morphology of the median nerve (MNCSA, circumference, D1, and D2) was investigated. First, correlations between the measurements obtained by the CNNs and those obtained by manual annotation were investigated. The integration of Spearman’s correlation coefficient (*r_s_*) revealed a variety of correlation intensities, as described in Table 2. Coefficient (*r_s_*) values of 0.2 ≤ *r_s_* ≤ 0.39, 0.4 ≤ *r_s_* ≤ 0.59, and 0.6 ≤ *r_s_* ≤ 0.79 were interpreted as weak, moderate, and strong relationships, respectively. According to our findings, SegNet and manual annotation had a significant weakly to moderately monotonic relationship in the extraction of morphological variables, whereas U-Net and manual annotation had a stronger significant monotonic relationship.

**Figure 6:**
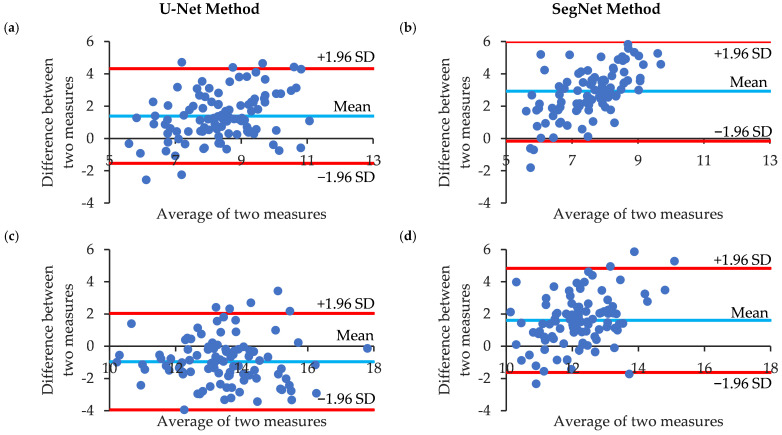

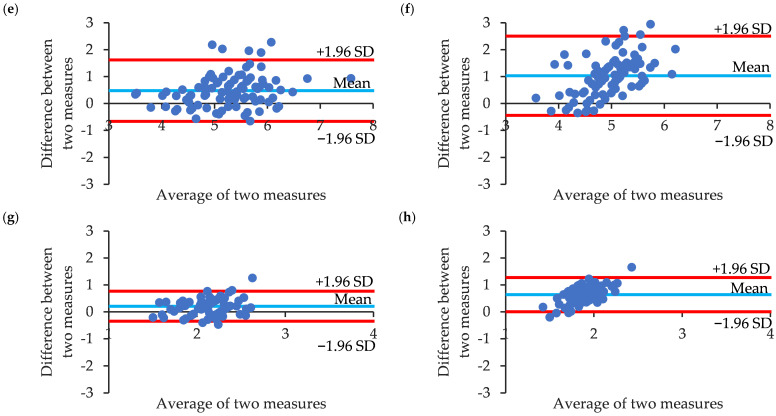
Bland–Altman analysis of the median nerve morphological parameters of manual annotation and CNN estimation. (**a**,**b**) Cross-sectional area (MNCSA); (**c**,**d**) circumference; (**e**,**f**) longitudinal diameter (D1); and (**g**,**h**) vertical diameter (D2).

Next, the agreement between the MNCSA, circumference, D1, and D2 results of the CNNs and manual annotations was investigated using Bland–Altman analysis. Bland–Altman analysis (*n* = 100) revealed that the average differences in the U-Net and SegNet results for the MNCSA were 1.39 and 2.93 (Figure 6a,b) and −0.96 and 1.61 for the circumference (Figure 6c,d), respectively. For the median nerve diameters, D1 had average differences of 0.48 and 1.03, and D2 had average differences of 0.21 and 0.64 (Figure 6e–h). The U-Net estimations were closer to the manual annotations. In addition, SegNet underestimates more measurements than does U-Net. Only the median nerve circumference was underestimated by U-Net. Although we observed measurement bias between the manual annotation and CNN results, more than 95% of the data were within the limits of agreement (mean difference ± 1.96 times the S.D. of the differences). Despite these differences, our findings indicate substantial congruency between the two models’ estimations.

## 4. Discussion

In this study, we compared the median nerve morphology between manual annotation and CNN models, namely, U-Net and SegNet. First, the learning curves of both U-Net and SegNet demonstrate that they can converge without overfitting. Both models achieved stability at approximately 20 epochs and exhibited similar loss histories. U-Net obtained substantially better precision, whereas SegNet’s recall results were slightly lower. As a result, U-Net outperformed SegNet in terms of DICE, primarily because of its superior precision.

Although U-Net consistently achieved a higher mean IoU, SegNet obtained variable results. Some images resulted in an IoU of <0.6, particularly when the median nerve presented an elongated morphology and a blurred outline. This highlights the need for model refinement to enhance the applicability and robustness of CNNs in medical US image analyses.

Both U-Net and SegNet demonstrated promising performance in terms of US image segmentation. In this study, all U-Net and SegNet output images had IoUs greater than 0. This shows that the CNN outputs and ground-truth images overlapped and that the CNNs were perfectly able to estimate the location of the median nerve. Owing to its symmetrical expansive path, U-Net provides effective and precise biomedical image segmentation. SegNet’s encoder–decoder architecture is known to offer reliable segmentation, particularly for a variety of non-medical road and infrastructure scenes. This demonstrates their individual performance abilities and applicability to semantic segmentation. Hence, researchers must carefully compare the merits of each approach, considering their applicability, learning efficiency, and segmentation accuracy in relation to the research area.

US imaging of the carpal tunnel is highly challenging because it relies heavily on skills such as the probe angle and pressure, which vary greatly across different US systems and often suffer from inconsistent noise levels that may result in poor image quality. Additionally, identification of the structures of the carpal tunnel, such as the wrist bones, TCL, finger flexor tendons, and median nerve, is necessary. Subsequently, most studies have reported manual annotation for the morphological evaluation of the median nerve [23,25]. Although good intra- and interrater reliabilities have been reported [28,37,38], a more accurate and objective image analysis method could support the process of obtaining more reliable data. Our results (Table 2 and Figure 6) suggest that the two methods are in good agreement.

This study has several limitations. First, our protocol involved the random selection of images from the dataset and included data from only healthy individuals, which could contribute to potential biases. Therefore, our protocol may not be directly applicable to the imaging analysis of acute or chronic CTS symptoms. Furthermore, because the ground-truth images were annotated manually, the examiner’s subjectivity may have an impact on the intra-rater accuracy and reliability. CNN methods for CTS image analysis, such as SegNet and U-Net, have shown promise. These CNN techniques can be improved with more varied image quality data to yield more accurate and efficient identification and estimation of median nerve features. These characteristics will make these methods more appropriate for use in future studies.

## 5. Conclusions

In this study, we examined the viability of median nerve morphology estimation using U-Net and SegNet. According to our findings, both models perform well in terms of precision, recall, DICE, and IoU. The application of both models to peripheral nerve imaging for dynamic or entrapment symptoms is promising. Future studies should investigate the sex, age, and biomechanical factors affecting the morphology of the median nerve.

## Figures and Tables

**Table 1:** Segmentation accuracies of U-Net and SegNet.

	U-Net	SegNet
**Precision**	0.811 ± 0.095	0.677 ± 0.114
**Recall**	0.869 ± 0.077	0.897 ± 0.084
**DICE**	0.833 ± 0.053	0.765 ± 0.081
**IoU**	0.717 ± 0.074	0.625 ± 0.099

**Table 2:** Spearman’s rank correlation between the results of manual annotation and the CNNs (*n* = 100).

	CNN	
Measurements	U-Net	SegNet
Median nerve cross-sectional area (MNCSA)	*r_s_* (98) = 0.517, *p* < 0.001	*r_s_* (98) = 0.337, *p* = 0.001
Circumference	*r_s_* (98) = 0.424, *p* < 0.001	*r_s_* (98) = 0.233, *p* = 0.020
Diameter (Longitudinal, D1)	*r_s_* (98) = 0.606, *p* < 0.001	*r_s_* (98) = 0.317, *p* = 0.001
Diameter (Vertical, D2)	*r_s_* (98) = 0.440, *p* < 0.001	*r_s_* (98) = 0.061, *p* = 0.546

**Figure A1:**
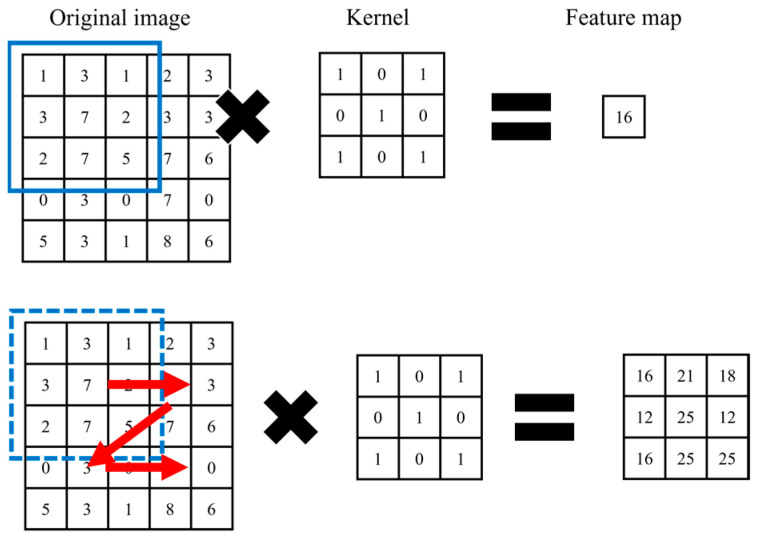
Example of convolution.

**Figure A2:**
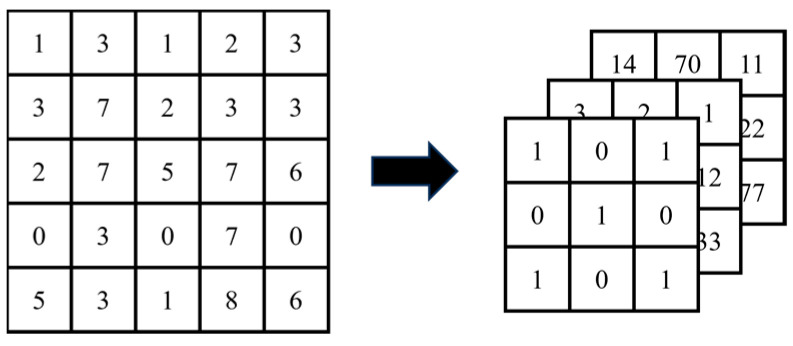
Convolution with multiple kernels.

**Figure A3:**
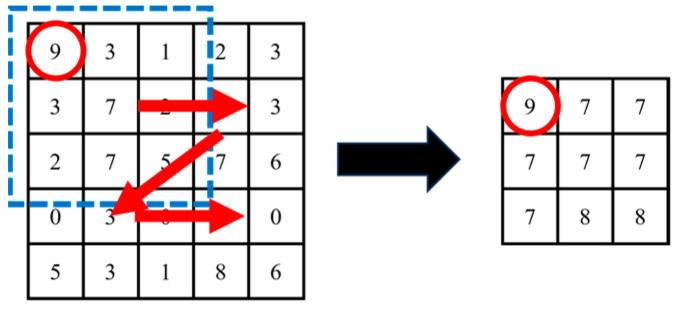
Example of max pooling.

**Figure A4:**
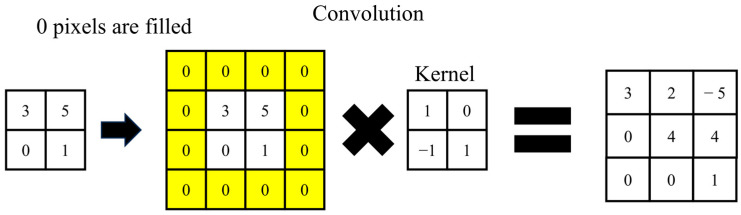
Example of deconvolution.

**Figure A5:**
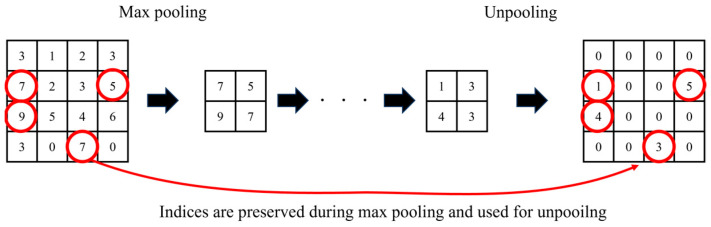
Example of unpooling.

**Figure A6:**
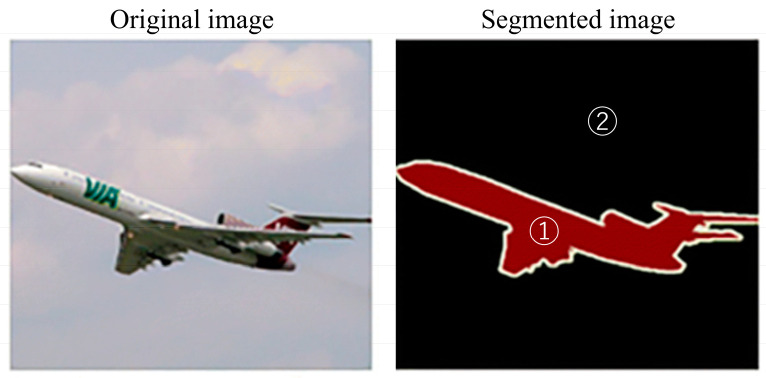
Example of semantic segmentation.

**Figure A7:**
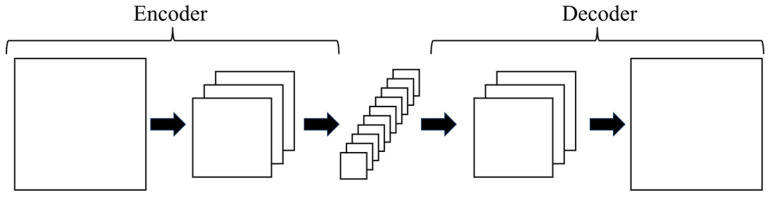
Architecture of encoder–decoder.

**Figure A10:**
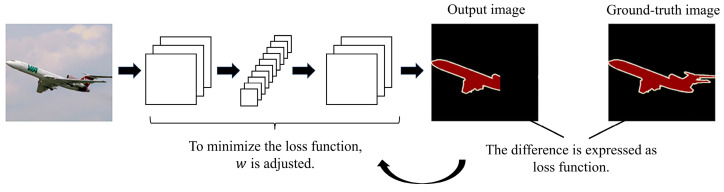
Schematic of learning process.

**Figure A11:**
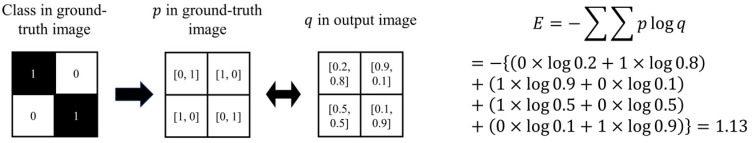
Example of binary cross-entropy.

## Data Availability

The data that support the findings of this study are available from the corresponding authors upon reasonable request.

## Appendix A. U-Net and SegNet Architectures

This study is multidiscipline, and the background of readers is expected to be diverse. While the CNN used in this study is well known for data scientists, it is unfamiliar for some clinicians and medical professionals. This appendix explains the CNN and related jargon to support the understating of such readers. Since the research area of the CNN is broad, it is impossible to cover all knowledge in this paper. Thus, this appendix tries to explain the CNN as simply as possible and is not algebraically accurate.

1. Convolution

Convolution is expressed as

(A1) $$g\left(x,y\right)=w\times f\left(x,y\right)$$

where f(x,y) and g(x,y) are the pixel values of the original image and feature map, respectively, and w is the weight of the filter (it is called a kernel). Figure A1 shows an example. By calculating Equation (A1), a feature map is obtained. Because the size of the original image and kernel is 5 × 5 and 3 × 3, respectively, the feature map is 3 × 3 in size. Thus, by performing convolution, the size of the image can be reduced. Moreover, several feature maps can be produced by preparing different kernels (Figure A2). In addition, a smaller feature map is produced by repeating convolution. In the learning process of deep learning, w is optimized to minimize the loss function (see 8. Binary cross-entropy).

2. Pooling

As well as convolution, pooling is performed to obtain the feature map with reduced size. Figure A3 shows the schematic of pooling. As an example, max pooling is applied in Figure A3, where the largest values in the filter are derived.

3. Deconvolution and unpooling

Deconvolution and unpooling are similar to the reverse processes of convolution and pooling. These are performed to increase the size of the feature map. Figure A4 and Figure A5 show examples.

Deconvolution is also called transposed convolution because deconvolution is not accurately the reverse of convolution. In deconvolution, after filling the vicinity of the feature map with pixels of 0, convolution is applied (Figure A4). On the other hand, unpooling is performed to increase the size of the feature map by filling 0 pixels. By preserving the indices during the max pooling, the created feature map tries to reproduce the original image as accurately as possible.

4. Semantic segmentation

Semantic segmentation is an image processing to categorize each pixel in an image. Figure A6 shows an example after semantic segmentation, the pixel in class ① and ② belongs to the aircraft and background, respectively.

5. Encoder–decoder

The convolutional neural network (CNN) is one of the deep learning algorithms. The CNN constitutes repeated downsizing and upsizing processes such as convolution, deconvolution, and pooling.

One of the CNN architectures is an encoder–decoder (Figure A7). In the encoder, the feature map with small size is produced to derive important features from the original image. In the decoder, on the other hand, the output image is produced by upsizing the feature map.

6. U-Net

U-Net has the encoder–decoder architecture (Figure A8). The encoder constitutes convolution and max-pooling layers and produces a feature map with a size of 8 × 8. Each encoder block includes one max pooling and two or three convolution layers, whose kernel size is 3 × 3 and 2 × 2, respectively. On the other hand, the decoder constitutes deconvolution and convolution and produces an output image with a size of 256 × 256.

The unique characteristic of U-Net is the skip connection between the encoder and decoder. During the downsizing process in the encoder, some information is lost. By copying the feature map in the encoder and concatenating to the decoder, the loss is reduced.

7. SegNet

As well as U-Net, SegNet has an encoder–decoder architecture. It produces an 8 × 8 feature map through an encoder. Each block contains two or three convolutional layers and one max-pooling layer, and their kernel sizes are 3 × 3 and 2 × 2, respectively. Unlike U-Net, SegNet preserves the pooling indices during max pooling, thereby allowing unpooling in the decoder. This reduces the number of parameters required for deconvolution, reducing the calculation time and memory capacity. Instead, because SegNet does not have skip connections, it could lead to the loss of pixel details.

8. Binary cross-entropy

An output image is produced through the U-Net or SegNet. During the learning process of U-Net and SegNet, w is adjusted to produce an output image which resembles the ground-truth image (Figure A10). The difference between the output and ground-truth image is expressed as a loss function. Thus, it can be said that the objective of learning is to adjust w to minimize the loss function.

One of the most general loss functions is the binary cross-entropy and is defined as

$$E=\sum_{n=1}^{N}\sum_{k=0}^{1}p{\left(x_{n}\right)}_{k}\log q{\left(x_{n}\right)}_{k}$$

where n is the pixel, k is the class, and $p{\left(x_{n}\right)}_{k}$ and $q{\left(x_{n}\right)}_{k}$ are the true and expected probability distributions, respectively. Let us think about the ground-truth image where every pixel belongs to class 0 or 1 (Figure A11). Each pixel in the output image has a probability distribution. E in Figure A11 is calculated as 1.13.

9. ReLU and Adams

In the convolution layer, ReLU is performed in Figure A8 and Figure A9. ReLU stands for the rectified linear unit and is expressed as

$$f\left(u\right)=max\{0,u\}$$

where u is the input. In other words, when the pixel value, u, is negative, the output is 0. On the other hand, when u is positive or 0, the output is not varied.

The weights w are adjusted to minimize E during the learning process. This adjustment was performed by calculating ∇E(w)=0. Due to its nonlinearity of E, some problems occur. One of the problems is the vanishing gradient problem, where the gradient of the function approaches 0 with the increase in the number of layers. To solve this problem, ReLU is usually applied.

The equation ∇E(w)=0 cannot be solved directly. In many cases, w is calculated by iteration. Thus, how to update w, i.e., how to calculate $w^{\left(t+1\right)}$, is important (t is iteration step). Although many calculation methods of $∆w(=w^{t+1}-w^{t})$ have been proposed, the most general one is Adam. It is defined as

$$∆w=-\eta \frac{m_{t}}{\sqrt{v_{t}+\epsilon }}$$

$$m_{t}=\rho_{1}m_{t}+\left(1-\rho_{1}\right)\nabla E\left(w^{t}\right)$$

$$v_{t}=\rho_{2}v_{t}+\left(1-\rho_{2}\right)\nabla E{\left(w^{t}\right)}^{2}$$

where η, $\rho_{1}$, $\rho_{2}$, and ϵ are user-defined parameters.
